# Nuclear TFE3 expression is a diagnostic marker for Desmoid-type fibromatosis

**DOI:** 10.1186/s13000-019-0814-4

**Published:** 2019-05-01

**Authors:** Luting Zhou, Haimin Xu, Jun Zhou, Lei Dong, Peipei Zhang, Xiaoqun Yang, Chaofu Wang

**Affiliations:** Department of Pathology, Ruijin Hospital, Shanghai Jiaotong University School of Medicine, Shanghai, 200025 China

**Keywords:** DTF, TFE3, Differential diagnosis, Immunohistochemistry

## Abstract

**Background:**

Desmoid-type fibromatosis (DTF) is a lesion characterized by clonal proliferation of myofibroblasts, which exhibits an infiltrative growth pattern. It is necessary for them to be distinguished from other fibroblastic and myofibroblastic lesions as well as spindle cell tumors. Altered Wnt signaling can act as a defining characteristic of DTF, with nuclear β-catenin serving as a diagnostic marker for. Transcription factor E3 (TFE3) has been linked to Wnt pathway activation and regulation, and may add value to the diagnosis of DTF. The present study, therefore, sought to assess whether TFE3 is a specific diagnostic marker for DTF.

**Methods:**

Nuclear TFE3 and β-catenin staining was performed on a wide range of tumor types such as DTF (*n* = 46), nodular fasciitis (*n* = 14), neurofibroma (*n* = 5), dermatofibrosarcoma protuberans (*n* = 5), gastrointestinal stromal tumor (*n* = 10), sclerosing epithelioid fibrosarcoma (*n* = 2), synovial sarcoma (*n* = 5), leiomyoma (*n* = 3) and cutaneous scar tissue (*n* = 4) using an immunohistochemical approach. In addition, the clinicopathological features and localization of these tumors were summarized. FISH assay was carried out to examine Xp11.2 translocations/TFE3 gene fusions. Statistical difference between immunohistochemical expression of TFE3 and β-catenin was analyzed.

**Results:**

The expression of nuclear TFE3 protein was found in 43 (93.5%) DTF tissue samples, ranging from moderate to intense expression levels. The distribution rates of TFE3 positivity in nodular fasciitis, gastrointestinal stromal tumor, leiomyoma and scar tissue samples were 42.9, 40, 25 and 33%, respectively. All studied samples of neurofibroma, synovial sarcoma, sclerosing epithelioid fibrosarcoma and dermatofibrosarcoma protuberans were negative for TFE3.

**Conclusions:**

This study reveal that TFE3 has a potential to serve as a diagnostic marker capable of assisting in the differential diagnosis of DTF and other spindle cell lesions.

## Background

Desmoid-type fibromatosis (DTF) is a form of aggressive localized fibroblastic or myofibroblastic tumor that occur in deep soft tissues, with infiltrative but non-metastatic growth [1, 2]. DTF remains relatively rare, making up just 0.03% of all neoplasms, which affects individuals between the ages of 15 and 60 years [3]. DTF patients aged 40 and below are primarily women, and the tumors are most commonly located at the abdominal wall [3]. DTF can, however, arise in all anatomical regions, and is often divided into intra-abdominal, abdominal and extra-abdominal types [4]. DTF lesions at intra-abdominal sites are primarily linked with familial adenomatous polyposis (FAP) or Gardner syndrome, with 13% of patients having one of these two conditions [5]. Pathological diagnosis of DTF is typically based on the presence of infiltrating lesion, microscopy examination and immunophenotyping. DTF lesions show a uniform spindle structure with abundant collagen upon microscopic examination. In addition, up to 75% of DTF lesions are positive for nuclear β-Catenin and such staining is often used for the differential diagnosis of DTF in order to distinguish it from other benign or malignant fibroblastic and myofibroblastic lesions, or gastrointestinal stromal tumors (GIST) [6, 7].

DTF lesions have distinct genetic alterations, such as mutations in β-catenin encoding gene CTNNB1, unlike those present in other forms of fibroblastic and myofibroblastic lesions [8]. β-catenin is an adhesion factor that plays a crucial role in Wnt signaling, and the downstream Wnt signaling molecule LEF (lymphoid enhancer-binding factor) has been used to differentiate between DTF, GIST and sclerosing mesenteritis [9]. DTF CTNNB1 mutations most frequently occur in exon 3 of this gene, which protect β-catenin from degradation via adenomatous polyposis coli (APC) protein complex and thus enhance β-catenin nuclear accumulation [10]. Stabilized β-catenin is translocated to the nucleus, where it binds to LEF transcription factors, and subsequently inactivates corepressors and recruits additional coactivators to Wnt target genes, leading to activation of TEF pathway.

Transcription factor E3 (TFE3) plays important roles in autophagy, lysosomal biogenesis and cellular stress responses [11], and serves as an oncogene in many tumor types with rearrangement at Xp11.2, including renal cell carcinomas, alveolar soft tissue sarcomas and perivascular epithelioid cell neoplasms [12, 13]. TFE3 can also activate Wnt/β-catenin signaling, creating a positive-feedback loop between this MITF family member and the Wnt signaling pathway. MITF can also enhance Wnt signaling through GSK3 or Axin, facilitating MITF stabilization [14, 15]. Recently, TFE3 has been used as a diagnostic marker for solid pseudopapillary neoplasms of the pancreas [16].

At present, the association between TFE3 expression and DTF development remains largely unknown. It is hypothesized that TFE3 may be a useful diagnostic marker for DTF, and thus we sought to assess the expression of TFE3 in 46 DTF cases via immunohistochemical approach. In addition, we compared the resultant TFE3 expression patterns with other tumor types to evaluate the specificity of this marker for DTF lesions.

## Methods

### Patient samples

A total of 46 surgically resected DTF specimens were collected at the archives of the Ruijin Hospital in Shanghai, China. The samples were collected between January 2014 and August 2017. To assess the marker specificity for DTF growth, we also obtained the samples of nodular fasciitis, neurofibroma, dermatofibrosarcoma protuberans (DFSP), gastrointestinal stromal tumor, sclerosing epithelioid fibrosarcoma, leiomyoma, low grade fibromyxoid sarcoma, and cutaneous scar tissues. All specimens were fixed with 10% formaldehyde and embedded in paraffin. Sections were cut at 4 μm thickness, and then stained with hematoxylin and eosin.

### Immunohistochemical staining

The 4-μm-thick sections were subjected to immunostaining with a TFE3 antibody (ZA-0657; ZSGB-BI) and a β-catenin antibody (IR702; Dako) using the Dako Omnis automated staining platform. TFE3, a monoclonal antibody was diluted to a ratio of 1:100. Animal serum and buffer were used as a negative control instead of primary antibody. Immunoreaction of TFE3 was performed on the DAKO automated immunostainer. The antibody was optimized using Ventana DAB detection kit (Ventana Medical Systems) and standard quality control procedures were carried out. The detection of TFE3 was performed following heat-induced antigen retrieval using ER2 antigen retrieval buffer. Xp11.2 renal cell carcinomas were used as positive control, while negative cell controls were also used.

### Evaluation of immunohistochemical staining

Two pathologists reviewed immunohistochemistry (IHC) staining results, assessing only nuclear TFE3 and β-catenin positivity for each tissue section. Quantitative analysis of TFE3 expression was conducted on the basis of staining extent and intensity as follows: 3 (strong staining), 2 (moderately intense staining), 1 (weak staining), and 0 (negative). The percentage of TFE3-positive nuclei was recorded in 10% increments from 0 to 100% and were scored as follows: 3 (> 70%), 2 (40–70%), 1 (10–40%), and 0 (< 10%). A histologic score was then obtained by multiplying these two scores together, and the resultant immunopositivity scores were classified as follows: 3+ (a score of 9), 2+ (a score of 4 or 6), 1+ (a score of 2 or 3), and negative (a score of 0 or 1).

### Fluorescence in situ hybridization (FISH) analysis

To assess Xp11.2 translocations/TFE3 gene fusions, FISH assay was conducted with a Zytolight SPEC TFE3 Dual color break-apart probe. All specimens were fixed in 10% neutrally buffered formalin for 24 h at room temperature (18–25 °C), while 4-μm-thick sections was fixed for 2–16 h at 50–60 °C. Sample pretreatment (dewaxing, proteolysis) was performed according to the instructions for use of the ZytoLight FISH-Tissue Implementation Kit. Briefly, 10 uL of the probe was added per tissue sample on a coverslip. Subsequently, the samples were denatured at 75 °C for 10 min, transferred to a humid chamber, and hybridized overnight at 37 °C. It is essential that specimens do not dry out during the hybridization step. FISH signals were detected under an Olympus BX51TRF microscope (Olympus, Japan) using a triple-pass filter (DAPI/Green/Orange; Vysis). The samples were considered FISH positive when the distance between red and green signals was ≥2 signal diameters.

### Statistical analysis

Statistical analysis of differences between DTF and other groups were performed using Mann-Whitney U test. The difference in immunohistochemical expression of TFE3 and β-catenin was analyzed by Chi-squared test using the SPSS software package. *P* value of less than 0.05 was considered to be statistically significant.

## Results

### Clinicopathologic findings

A total of 46 DTF tissue sections and 49 other lesion types (controls) were analyzed, with their clinicopathological features summarized in Table 1. Among the 46 DTF patients, 18 (39.1%) were male and 28 (60.9%) were female, with a mean age of 36 years (range 15–65 years). With regard to DTF localization, 16 (34.8%) were located in the abdominal wall, 15 (32.6%) in the abdominal cavity, 7 (15.2%) in the limbs, 7 (15.2%) in the back, chest and hips, and 1 (2.2%) in the breast. As compared to the control lesions used in this study, a higher frequency of DTF lesions was found among women. Among the control samples, nodular fasciitis was primarily presented in the limbs (73.3%), and all GIST and sclerosing epithelioid fibrosarcoma (SEF) occurred at the abdominal cavity.

**Table 1:** Patient clinicopathologic data

	DTF (*n* = 46)	NF (*n* = 14)	Neurofibroma (*n* = 5)	Leiomyoma (*n* = 3)	GIST (*n* = 10)	DFSP (*n* = 5)	IMT (*n* = 2)	SS (*n* = 5)	SEF (*n* = 2)	Scar (*n* = 4)
Age(y)	15–65	22–65	55–73	49–68	36–72	19–47	27–29	47–72	34–49	10–56
Mean(y)	36	40	65.2	57.3	55.4	34.4	28	55.7	41.5	28.8
male	18	6	3	1	7	1	2	3	1	1
female	28	9	2	2	3	4	0	2	1	3
Location										
Abdominal wall	16	0	1	1	0	1	0	0	0	
Abdominal cavity	15	0	1	1	10	0	1		2	0
back	3	2	1	0	0	0	0	0	0	0
Chest	2	1	1	1	0	2	0	0	0	2
Hipshot	2	0	0	0	0	0	1	0	0	0
Limb	7	10	1	0	0	1	0	5	0	2
Breast	1	1	0	0	0	1	0	0	0	0

*DTF* Desmoid-type fibromatosis, *NF* Nodular fasciitis, *GIST* Gastrointestinal stromal tumor, *DFSP* dermatofibrosarcoma protuberans, *IMT* Inflammatory myofibroblastic tumor, *SS* synovial sarcoma, *SEF* Sclerosing epithelioid fibrosarcoma, *TFE3* Transcription factor E3

### Pathologic findings

**Fig. 1:**
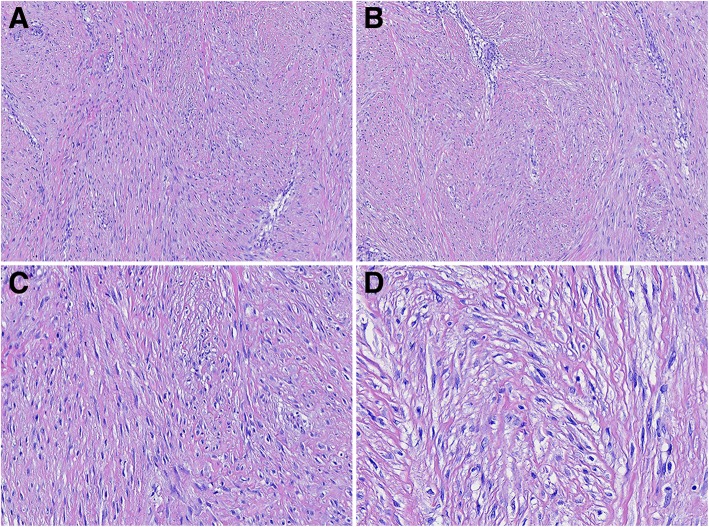
Histology of DTF samples. Tumors were poorly circumscribed, with evidence of local soft tissue infiltration. The samples were consisted of proliferating spindle-shaped fibroblasts, myofibroblasts and collagen (**a**, 100×). Fibroblasts were sparse or vacuolar (**b**, 100×). Cells lacked nuclear hyperchromasia and were arranged in long sweeping bundles (**c**, 200× and **d**, 400×)

The 46 DTF cases exhibited clear or unclear boundaries. The largest tumor was 26 cm in diameter and located in the posterior peritoneum, whereas the smallest was 1.5 cm in diameter and located in the upper arm. Upon visual inspection, these lesions appeared gray-white, gray-red and tough. Under light microscopy, DTF sections displayed unclear peripheral regions with an evidence of infiltration, and were composed of spindle fibroblasts, myofibroblasts and collagen fibers. Sparse chromatin was observed in these fibroblasts and myofibroblasts, with small nucleoli and little evidence of mitosis. Moreover, the cells were arranged in parallel bundles. In some instances, abdominal DTF samples showed a uniform arrangement that was difficult be distinguished from GIST based on cellularity alone. In other instances, DTF samples were wavy in shape, which can be easily misdiagnosed as neurofibroma based on such morphological character. Besides, in other DTF samples, myxoid degeneration was presented, leading to a considerable difficulty in distinguishing these samples from low-grade malignant fibromyxoid sarcomas (Fig. 1).

### Immunohistochemical results

**Fig. 2:**
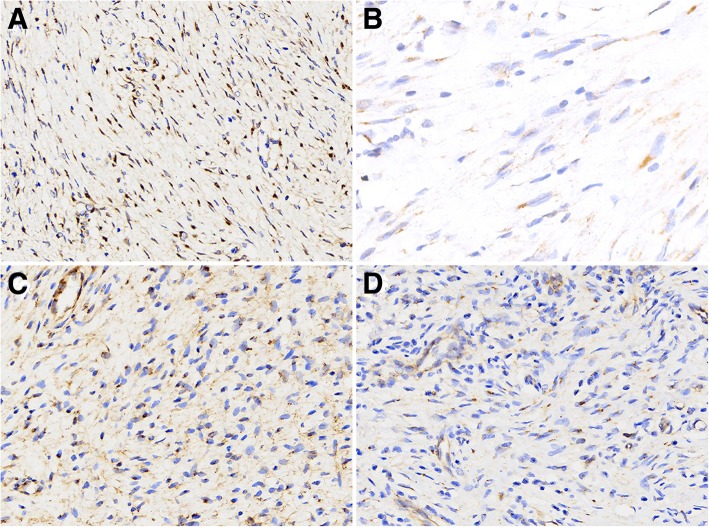
DTF samples demonstrated positive nuclear β-catenin staining (**a**, 400x), while NF, neurofibroma and DFSP samples were all negative for nuclear β-catenin (**b**, **c** and **d**, 400×)

Immunohistochemical staining of β-catenin was conducted on all 46 DTF, 15 NF (nodular fasciitis), 5 neurofibroma, 3 leiomyoma, 10 GIST, 5 DFSP, 2 inflammatory myofibroblastic tumor, 5 synovial sarcoma, 2 SEF, and 4 scar tissue samples. Approximately 82.6% (38/46) of DTF cases showed positive nuclear β-catenin staining, while other control tissues demonstrated a lack of nuclear β-catenin expression (Fig. 2).

**Fig. 3:**
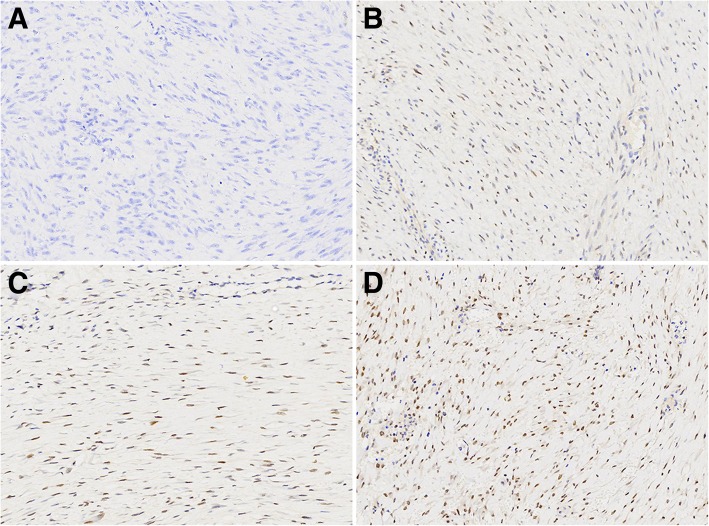
Variations of TFE3 expression in DTF. Negative (**a**, 400×), weak positive (**b**, 400×), moderate positive (**c**, 400×), and strong positive (**d**, 400×) expression of TFE3 in DTF

**Fig. 4:**
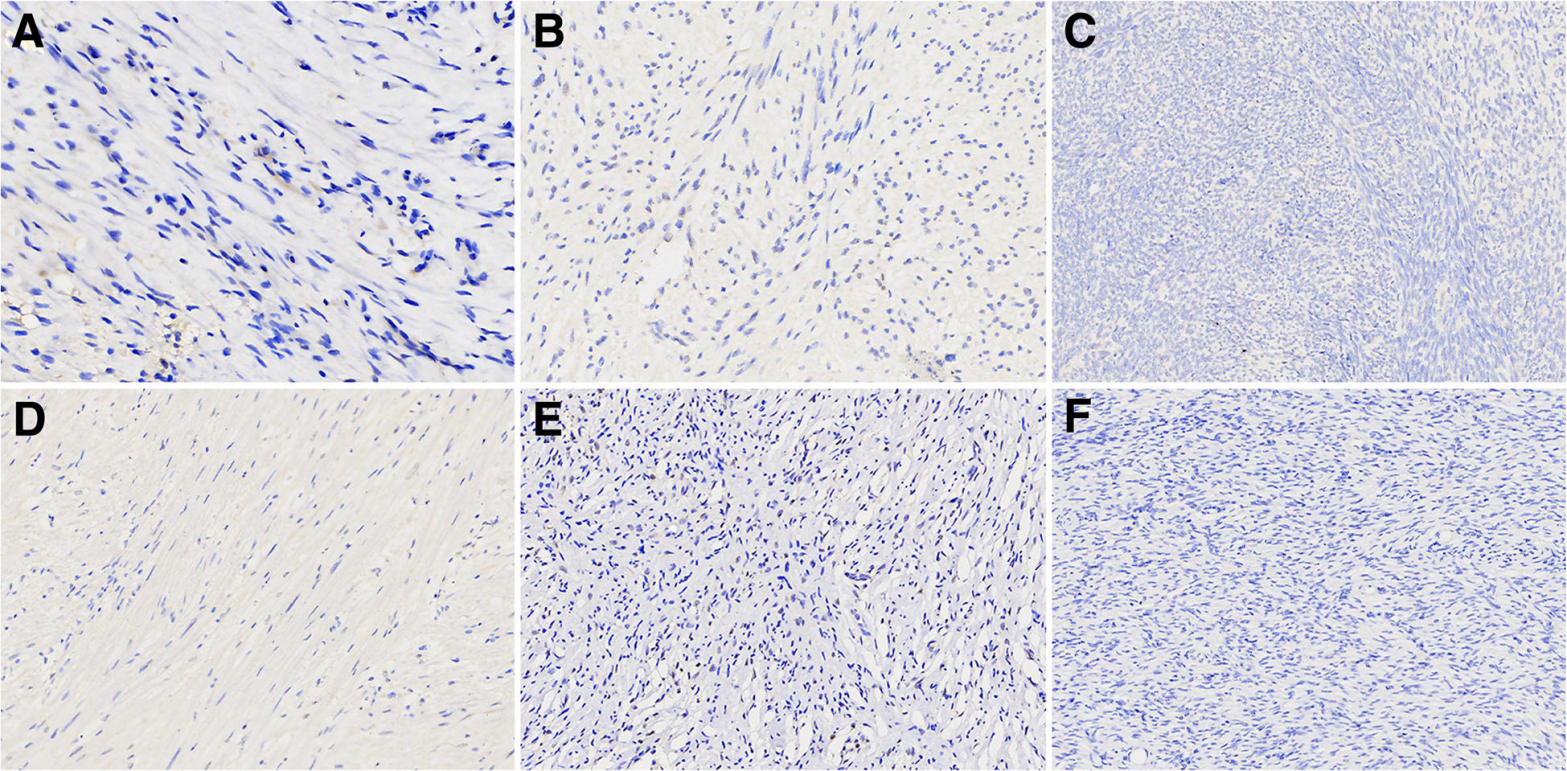
TFE3 expression in other fibroblastic and myofibroblastic lesions and spindle cell tumors. NF exhibited TFE3 negative expression (**a**, 400×), GIST showed TFE3 negative expression (**b**, 400×), while synovial sarcoma (**c**, 400×), leiomyoma (**d**, 400×), neurofibroma (**e**, 400×), and DFSP (**f**, 400×) were all negative for TFE3

**Fig. 5:**
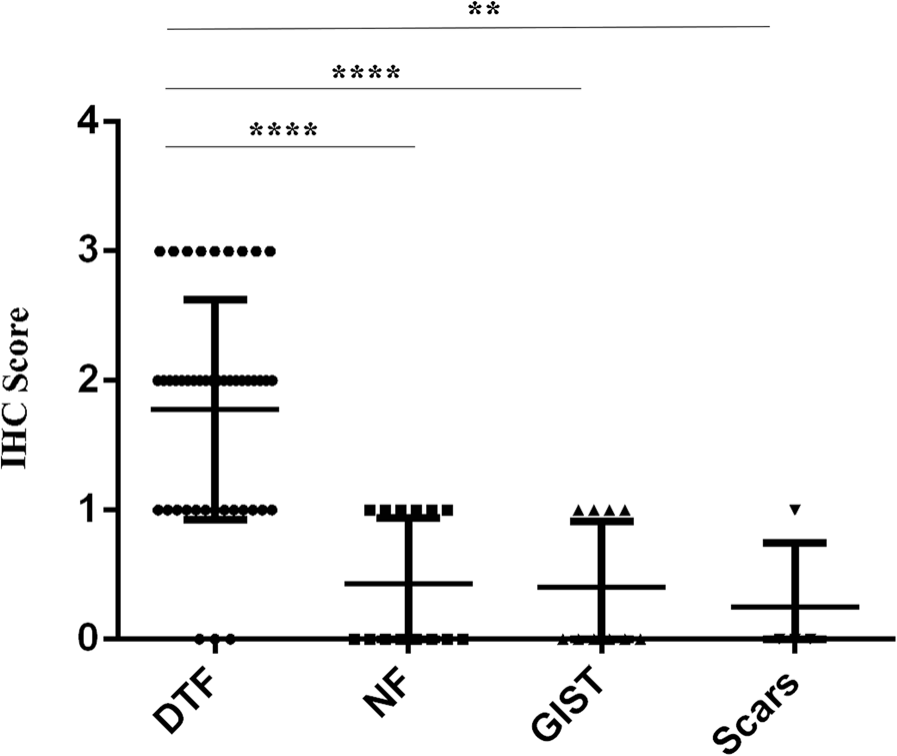
Immunohistochemical results of TFE3 expression in DTF, NF, GIST and scars tissue samples. Quantitative analysis of TFE3 expressions were revealed by immunohistochemical score. Data are presented as mean ± SEM. Levels of significance between DTF and other groups were calculated by Mann-Whitney U test (** *P* < 0.01 and **** *P* < 0.0001)

Immunohistochemical staining of TFE3 was performed on all 46 DTF samples, of which 43 (93.5%) were positive for TFE3 expression and 3 (6.5%) were negative. Notably, the immunoreactivities of TFE3 appeared to be weakly positive (1+) in 13 (28.3%) cases, moderately positive (2+) in 20 (43.4%) cases, and strongly positive (3+) in 10 (21.7%) cases (Fig. 3). Among the control group (14 NF, 4 cutaneous scar, 10 GIST, 3 leiomyoma, 5 neurofibroma, 5 synovial sarcoma and 5 DFSP), 6 (42.9%) NF samples were weakly positive for nuclear TFE3 (1+) immunoreactivity, and the rates of nuclear TFE3 positivity in cutaneous scars and GIST were 25 and 40%, respectively. All the examined cases of neurofibroma, leiomyoma, sclerosing epithelioid fibrosarcoma, synovial sarcoma and DFSP were negative for TFE3 nuclear staining (Fig. 4). The overall results of TFE3 immunohistochemical staining in DTF and other lesions are summarized in Table 2, In addition, the IHC scores of TFE3 were significantly higher in DTF compared to those in NF, GIST and scar tissue samples (Fig. 5).

**Table 2:** TFE3 immunohistochemical staining results

	n	0	1+	2+	3+	Total(%)
DTF	46	3	13	20	10	93.5%
NF	14	8	6	0	0	42.9%
Neurofibroma	5	5	0	0	0	0
Leiomyoma	3	3	0	0	0	0
GIST	10	6	4	0	0	40%
DFSP	5	5	0	0	0	0
IMT	2	2	0	0	0	0
SS	5	5	0	0	0	0
SEF	2	2	0	0	0	0
Scars	4	3	1	0	0	25%

*DTF* Desmoid-type fibromatosis, *NF* Nodular fasciitis, *GIST* Gastrointestinal stromal tumor, *DFSP* dermatofibrosarcoma protuberans, *IMT* Inflammatory myofibroblastic tumor, *SS* synovial sarcoma, *SEF* Sclerosing epithelioid fibrosarcoma, *TFE3* Transcription factor E3

For the comparison of β-catenin and TFE3 positivity in DTF, 43 (93.5%) and 38 (82.6%) DTF cases were positive for TFE3 and β-catenin nuclear staining, respectively. Notably, 80.4% (37/46) of DTF cases were positive for both TFE3 and β-catenin, while 13% (6/46) were positive for TFE3 but not β-catenin. Only 1 (out of 46) cases was positive for β-catenin and negative for TFE3, while the remaining 2 cases were negative for both nuclear TFE3 and β-catenin staining (Table 3). In overall, the distribution of DTF cases with strong immunopositivity for TFE3 and β-catenin was significantly higher (*p* = 0.0199) compared to other groups, indicating the expression levels of TFE3 and beta-catenin are both correlated and consistent.

**Table 3:** Comparison of β-catenin and TFE3 Positivity in DTF

Results	TFE3		Total
	Positive	negative	
β-catenin			
Positive	37	1	38
Negative	6	2	8
Total	43	3	46
*P =* 0.0199			

*DTF* Desmoid-type fibromatosis, *TFE3* Transcription factor E3

### FISH results

**Fig. 6:**
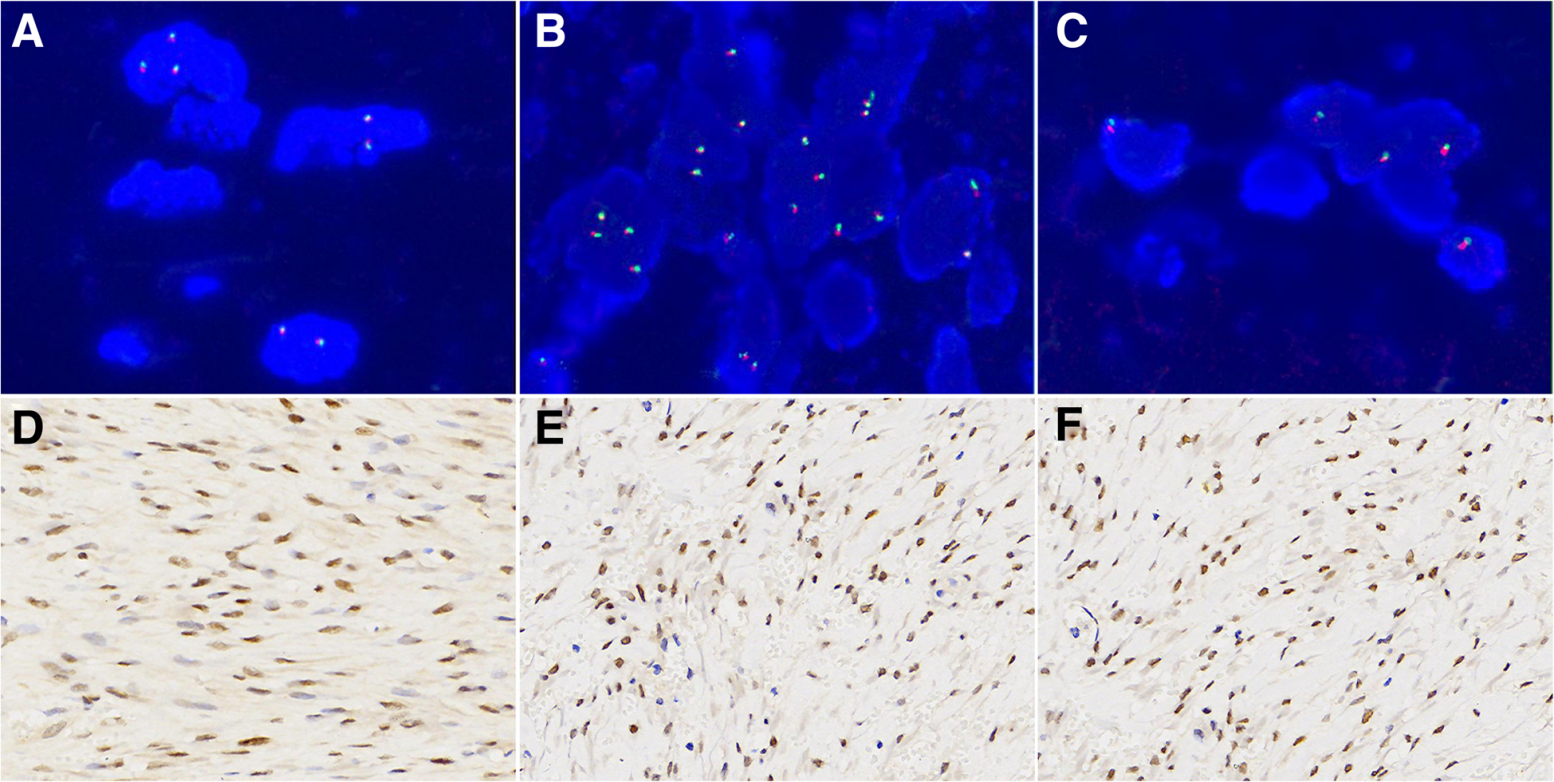
Representative images of TFE3 FISH staining. A TFE3 break-apart probe assay allowed for the visualization of normal fused hybridization signals (**a**, **b** and **c**, 1000×). In total, 3 DTF cases with strong positive TFE3 IHC staining were selected for this assay, and the results showed limited evidence of Xp11.2 rearrangement (**e**, **f** and **g**, 400×)

Next, three DTF samples with strong nuclear TFE3 were selected for FISH-based analysis. Through a break-apart FISH assay, we observed a combination of red and green signals that indicates lack of signal splitting. A total of 2.0, 2.7 and 3.0% rearrangement-positive cells were detected in the three cases, as revealed by FISH analysis (Fig. 6). These results suggest the absence of TFE3 gene rearrangement in DTP cases.

## Discussion

DTF is a form of low-grade soft tissue tumor that often difficult to be distinguished from more severe tumors, owing to its morphological and immunohistochemical similarities with other benign or malignant spindle cell lesions. Aberrant nuclear β-catenin accumulation and CTNNB1 exon 3 mutations have been identified, with positive nuclear β-catenin staining found in 82–100% DTF cases [17–19]. The exact molecular and genetic basis of DTF are yet to be fully elucidated. To date, trisomy 8, trisomy 20, APC, and β-catenin mutations have been recognized for their contributions to the development of DTF [20–22]. In the present study, TFE3 nuclear staining exhibited the same sensitivity for DTF (93.5%), as similar to β-catenin nuclear staining. Furthermore, FISH analysis confirmed that there was no relationship between nuclear TFE3 staining and TFE3 translocation. Altogether, these results may provide novel insights into the diagnosis, etiology, and molecular mechanisms of DTF.

The Wnt/β-catenin-dependent pathway remains the well-studied defining feature of DTF lesions. Wnt signaling plays pivotal roles in regulating tissue regeneration and cancer development, which can be activated by TFE3 expression [23]. MITF is closely linked with Wnt/β-catenin signaling, as it sequesters GSK3 and Axin1 which stabilize it and allow it to translocate to the nucleus where it promotes expression of many lysosomal genes, resulting in an expansion of multivesicular bodies (MVBS) and a consequent enhancement in Wnt signal, generating a positive feedback loop linked with melanoma progression. TFE3, one of the four members of the microphthalmia transcription factor family, is encoded by a gene located on the short arm of chromosome 11 and is approximately 14.78 kb in length. This gene is expressed throughout the body, and can regulate the expression levels of many genes by interacting with regulatory factors such as SMAD3, E2FE3 and LEF-1. Through these interactions, TFE3 may affect TGF-β production, cell growth, macrophage differentiation, and other major physiological processes. Translocations of TFE3 gene can lead to a significant increase in its protein levels [24, 25], and thus is associated with a certain type of cancer. For instance, xp11.2 TFE3 translocation gene fusions occur in a subset of patients with renal cell carcinoma, especially children and young females, as revealed by both FISH analysis and positive IHC staining for TFE3. Moreover, TFE3 gene fusions have been identified in patients with alveolar soft part sarcoma, epithelioid hemangioendothelioma and hepatic perivascular epithelioid cell tumors [26–28].

In the present study, we assessed the relationship between TFE3 and Wnt/β-catenin signaling in DTF tumors, hypothesizing that TFE3 could be highly expressed in these samples. Indeed, it was found that nuclear TFE3 protein was expressed in 93.5% (43/46) of paraffin-embedded DTF tissue sections, in which 65.2% (30/46) of cases exhibited moderate (2+) to strong (3+) nuclear TFE3 staining. Moreover, the results of FISH analysis confirmed that the increased TFE3 protein levels were unrelated to TFE3 gene fusion. It was noted that only 38 (82.6%) of 46 DTF samples demonstrated positive nuclear staining of β-catenin protein, indicating TFE3 is a more sensitive marker for DTF.

Concordance of TFE3 and β-catenin IHC score was observed in 80.4% (37/46) DTF cases, where 13% (6/46) and 2.2% (1/46) cases were only positive for TFE3 and β-catenin, respectively. The remaining 2 cases were negative for both nuclear markers. These results thus support a correlation between TFE3 and β-catenin, suggesting that these markers can be used in a complementary fashion for diagnosing DTF.

From a purely morphological notion, it can be difficult to differentiate DTF from other benign and malignant fibroblastic and myofibroblastic tumors or spindle cells lesions. NF is mainly composed of spindle myofibroblasts that often express a-SMA in a diffuse manner. TFE3 expression was detected in 14 NF tissue samples, of which only 6 (42.9%) displayed weak positive TFE3 expression. Notably, a significant difference was found between the frequencies of these two tumor types. Some samples of DTF are rich in collagenous fibers and appeared similarly to scar tissues, but only 1 out of 4 scar samples demonstrated weak TFE3 positivity in this study. Neurofibromas are benign peripheral nerve sheath tumors, and none of our studied neurofibroma samples (0/4) were stained positive for TFE3. Likewise, 0/3 leiomyoma samples were TFE3 positive, and the same trend (0/2) was observed for intermediate type myofibroblastic tumors. Up to 50–70% of GIST lesions are composed of spindle cells, making them difficult to be distinguished from DTF. In addition, we found that 4/10 GIST lesions showed weak TFE3 positivity, suggesting it can be a candidate marker for differentiating between these two tumor types along with CD117, Dog1 and CD34. DFSP is composed of cytologically uniform spindled tumor cells, and 0/5 of the studied DFSP samples were TFE3 positive, as similar to SEF samples (0/2). Taken together, our results indicate that TFE3 is a valuable marker for differentiating DTF from NF, GIST, neurofibroma, leiomyoma, DFSP, synovial sarcoma, SEF, and scar tissue samples.

## Conclusion

The majority of DTF cases showed evidence of increased nuclear TFE3 expression, suggesting a possible association between this gene and DTF pathogenesis. Nonetheless, further work is needed to confirm these findings in a larger cohort. In addition, we verified that the observed increases in TFE3 expression were unrelated to Xp11.2 translocation. In all, our results suggest that TFE3 may be useful for distinguishing DTF from other forms of spindle cell lesions.

## Abbreviations

APC: Adenomatous polyposis coli
DFSP: Dermatofibrosarcoma protuberans
DTF: Desmoid-type fibromatosis
GIST: Gastrointestinal stromal tumor
LEF: Lymphoid enhancer-binding factor
NF: Nodular fasciitis
SEF: Sclerosing epithelioid fibrosarcoma
TFE3: Transcription factor E3
